# Potential Involvement of Complement Activation in Kidney Vascular Lesions of Arterionephrosclerosis

**DOI:** 10.3389/fmed.2022.836155

**Published:** 2022-03-31

**Authors:** Xuejing Chen, Yu Wang, Xiaojuan Yu, Suxia Wang, Minghui Zhao

**Affiliations:** ^1^Renal Division, Department of Medicine, Peking University First Hospital, Institute of Nephrology, Peking University, Key Laboratory of Renal Disease, National Health and Family Planning Commission of the People’s Republic of China, Key Laboratory of Chronic Kidney Disease Prevention and Treatment, Ministry of Education, Beijing, China; ^2^Laboratory of Electron Microscopy, Pathological Centre, Peking University First Hospital, Beijing, China; ^3^Peking-Tsinghua Center for Life Sciences, Beijing, China

**Keywords:** complement, arterionephrosclerosis, hypertension, benign nephrosclerosis, C3d

## Abstract

**Background:**

Complement dysregulation has been implicated in the pathogenesis of malignant nephrosclerosis with typical pathological manifestation as thrombotic microangiopathy (TMA) in recent studies. The aim of the present study was to evaluate the potential role of complement activation in arterionephrosclerosis, the major pathological change in benign hypertensive nephrosclerosis.

**Methods:**

Patients with biopsy-proven arterionephrosclerosis from 2010 to 2018 in our center were retrospectively enrolled in the present study. The clinical data were retrieved from the medical chart record. The pathological changes of renal biopsy were semiquantitatively evaluated. The ratio of inner-/outer-luminal diameter of the arterioles was calculated to evaluate the degree of arteriosclerosis. Immunohistochemical staining of CD34 and CD68 was adopted to evaluate peritubular capillary (PTC) density and macrophage infiltration, respectively. Complement components, including C3d, C4d, C1q, and C5b-9, were detected by immunohistochemical staining in paraffin-embedded sections. IgM and albumin were detected by immunofluorescence staining in frozen renal tissues.

**Results:**

Fifty-two patients were enrolled. The mean age was 45.0 ± 12.7 years, with 39 (75%) males. The median duration of hypertension was 66 months (IQR: 24–138 months). A total of 950 arterioles were evaluated, with a mean ratio of the inner/outer luminal diameter of 0.43 ± 0.05. The ratio of the inner-/outer-luminal diameter correlated with eGFR (*r* = 0.341, *p* = 0.013), sclerotic/ischemic glomerular lesions (*r* = –0.364, *p* = 0.008) and PTC density (*r* = 0.426, *p* = 0.002). Seventy-four percent (703/950) of the evaluated arterioles had C3d deposition with various patterns and intensities. The percentage of C3d-positive arterioles ranged from 63.6 to 100.0% in each specimen. The ratio of the inner/outer luminal diameter of arterioles correlated with the intensity of C3d deposition (*r* = –0.174, *p* = 0.001). Infiltration of macrophages was observed around C3d-positive arterioles. The percentage of C3d-positive arterioles was correlated with macrophage infiltration in each specimen (*r* = 0.330, *p* = 0.018). Occasional C4d-positive staining on arterioles was observed with no deposition of C1q or C5b-9 in arterionephrosclerosis specimens.

**Conclusion:**

Our findings provide evidence for potential complement activation in the pathogenesis of vascular lesions in arterionephrosclerosis.

## Introduction

Arterionephrosclerosis is characterized by vascular lesions with fibromuscular thickening of the intima and media of small arteries and arterioles, which induces narrowing of the lumen (arteriolosclerosis) and/or hyalinosis (1). Long-standing uncontrolled hypertension has been considered a major risk factor for arterionephrosclerosis, which is traditionally called benign hypertensive nephrosclerosis and may slowly progress to end-stage kidney diseases (ESKD). In addition to the long-holding theory that pathological lesions are induced by mechanical stress on the vascular walls, the mechanisms that cause vascular injury in arterionephrosclerosis are not completely understood. Interestingly, some hypertensive patients may experience episodes of severe blood pressure elevation during their disease course with acute exacerbation of their kidney function, which is called malignant nephrosclerosis with pathological manifestations as thrombotic microangiopathy (TMA) (2–4). Complement components (C3c and C5b-9) were identified along the vasculature and/or the glomerular capillary wall in malignant nephrosclerosis, suggesting aberrant complement activation, especially alternative pathway (AP) activation, in the development of severe hypertension-related kidney injury (5, 6). Although the exact mechanism for the transition from high blood pressure to malignant nephrosclerosis remains unclarified (7), elevated plasma C3 was reported in humans with prehypertension and hypertension, and plasma C3 levels were associated with future blood pressure increases and the development of hypertension in a population-based longitudinal study (8).

The complement system is an important part of innate immunity to defend against pathogen infection and maintain host homeostasis. Three pathways (classical, alternative, and lectin) activate the complement system and thus lead to the formation of C3 convertases to cleave C3. This step is critical to lead to downstream effector functions such as the formation of anaphylotoxins (C3a and C5a), lysis of pathogens through the membrane attack complex (MAC) (C5b-9), and opsonization of pathogens (C3b, iC3b, and C3d). Generally, AP is a continuously active surveillance pathway with continuous production of C3b, which is tightly regulated to prevent damage to the self (9–12). Activated C3b can covalently attach to the surface of microorganisms and/or the cell membrane, interact with complement receptor 1 and is converted to iC3b by factor I and factor H, which is subsequently cleaved to C3d by factor I. As the final degradation product of C3, C3d subsequently interacts with its receptors on phagocytic and immune cells to enhance clearance when conjugated with various foreign and self-proteins, which bridges the innate and acquired immune response. Recently, accumulating evidence suggests that the complement system plays an active role in the pathogenesis of hypertension and hypertension-related target organ damage (13–15). In the present study, we examined the deposition of complement components in renal biopsy specimens of patients with arterionephrosclerosis. The association of complement deposition with pathological lesions was further analyzed.

## Materials and Methods

### Patients

**FIGURE 1:**
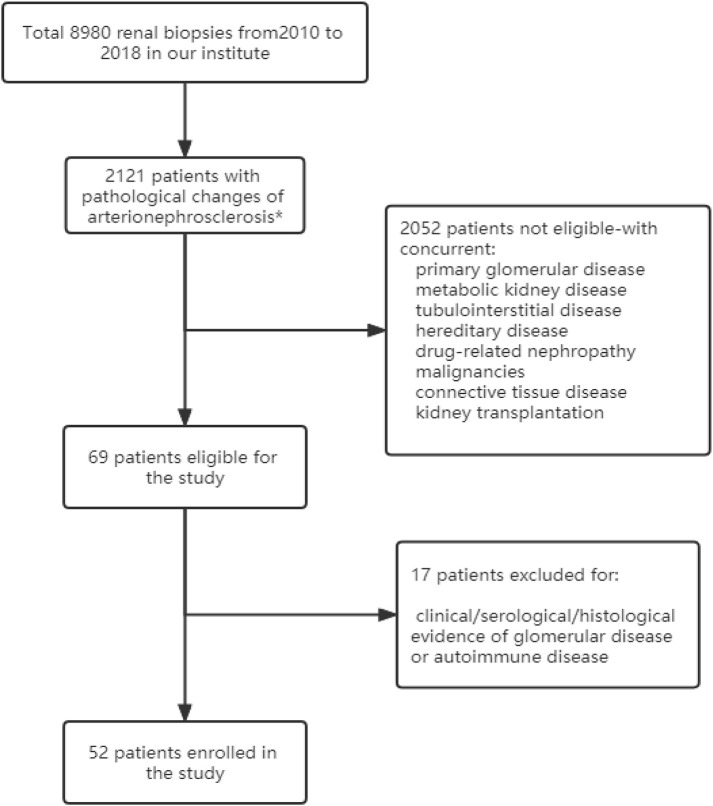
Flow chart of the study. *Vascular lesions with fibromuscular thickening of the intima and media of small arteries and arterioles, glomerulosclerosis, and IFTA (interstitial fibrosis and tubular atrophy).

This retrospective cohort study enrolled patients who underwent native renal biopsies at Peking University First Hospital from Jan 2010 to Dec 2018. Inclusion criteria: Patients with arterionephrosclerosis as the sole pathological diagnosis were eligible for enrollment. Renal biopsies were evaluated independently by at least two renal pathologists and/or nephrologists. Exclusion criteria: Patients with clinical, serological or histological evidence of concurrent kidney diseases such as glomerular disease, metabolic kidney disease, autoimmune disease, and tubulointerstitial disease were excluded (Figure 1). Five patients with malignant nephrosclerosis were used as disease controls. The diagnosis of malignant nephrosclerosis was defined as blood pressure ≥ 180/120 mmHg, grade III or IV hypertensive retinopathy according to the Keith-Wagener classification, and typical renal pathological features of thrombotic microangiopathy (TMA). Normal control kidney tissues were obtained from patients who received nephrectomy for tumors. Their medical records were reviewed to exclude previous hypertension history. Informed consent was obtained from each patient before biopsy. This study was approved by the ethics committee of Peking University First Hospital (2017[1280]). The research was conducted in accordance with the Declaration of Helsinki.

### Clinical Data

Demographic (age, sex), medical history (hypertension, diabetes, hyperuricemia/gout, hyperlipidemia, alcohol, and smoking), and biological data [body mass index (BMI), systolic blood pressure (SBP) and diastolic blood pressure (DBP)] were collected from electronic medical charts, as were laboratory data [serum creatinine (Cr), complement 3 (C3) and 4 (C4), 24-h urinary protein, urinary RBC count, serum albumin, hemoglobulin, platelet, and lactate dehydrogenase] at the time of renal biopsy. eGFR was calculated using the CKD-EPI equation. Severe bleeding complication of renal biopsy, which was defined as requiring intervention, including blood transfusion or an invasive procedure (radiological or surgical) due to bleeding was also recorded.

### Histologic Evaluation

The renal biopsy specimens were stained with periodic acid Schiff (PAS) reagent, periodic acid silver methenamine, hematoxylin–eosin, and Masson’s trichrome and examined using immunofluorescence, light microscopy and electron microscopy. Three types of glomerular lesions were identified: global glomerulosclerosis, segmental glomerulosclerosis and ischemic glomerular changes with thickening and wrinkling of the glomerular basement membrane. Two types of vascular lesions were identified: arteriolar hyalinosis and arteriosclerosis with thickening of the arteriole wall. Interstitial lesions included inflammatory cell infiltration, interstitial fibrosis and tubular atrophy (IFTA). The lesions were scored as follows for subsequent analysis:

1. Glomerular lesions were scored as the percentage of global/segmental glomerulosclerosis and ischemic glomerular change over total glomeruli in each specimen.
2. Arteriolar hyalinosis was scored as 0 (absent) and 1 (present).
3. Arteriosclerosis was evaluated by measuring the inner and outer diameters of the lumen separately on cross sections. A ratio of inner to outer luminal diameter less than 0.5 was deemed thickening of the arteriole wall. The arteriosclerosis value for each patient was expressed as the mean of the ratios of inner-/outer-luminal diameter of all arterioles observed in the specimen.
4. IFTA was scored as percentages of the involved area over total area analyzed on the specimen stained with Masson.

### Immunofluorescence Examinations for C3d, IgM, and Albumin

Five-micrometer-thick sections of frozen renal biopsy tissues were fixed in ice-cooled acetone for 10 min and then washed three times with PBS. Sections were incubated with primary antibody for C3d overnight at 4°C. After washing with PBS, the sections were incubated with FITC-conjugated secondary antibody (donkey anti-rabbit IgG, Jackson, United States) for 30 min at 37°C. Consecutive sections were incubated with FITC-labeled rabbit anti-human albumin and FITC-labeled rabbit anti-human IgM (both diluted 1:100 in PBS) at 37°C for 30 min, respectively.

### Immunohistochemistry Examinations

In brief, 4-μm-thick sections of formalin-fixed and paraffin-embedded biopsies were deparaffinized with xylene and ethanol. High-pressure treatment, proteinase K or 0.4% pepsin digestion was used for optimal antigen retrieval as instructed in the information sheet of each primary antibody. Endogenous peroxidase activity was blocked by incubation with 3% hydrogen peroxide for 10 min. After further blocking with 10% non-immune serum in phosphate-buffered saline (PBS) for 30 min, sections were incubated with primary antibodies overnight at 4°C. Information on the primary antibodies is listed in Supplementary Table 1. After washing with PBS, the sections were further incubated with biotinylated secondary antibody (Zhongshan Golden Bridge Biotechnology, Beijing, China) for 45 min at 37°C and incubated with diaminobenzidine chromogen for color development. Finally, the sections were counterstained with hematoxylin, dehydrated with graded alcohol and xylene, covered with HistoMount (Thermo Fisher Scientific) and sealed. Normal homologous serum was used to replace the first antibody as a negative control.

All sections were scanned by a slide scanner (Scanscope, Aperio) and analyzed by Image-Pro Plus software. The intensity for C3d deposition was evaluated at a magnification of 400 × and scored as a percentage of the positive staining area in relation to the total area of the arteriole wall analyzed. The density of peritubular capillaries (PTCs) was expressed as counting the number of PTCs in *per* total area analyzed in the specimen, with glomeruli excluded. Infiltration of macrophages was evaluated by counting CD68-positive cells *per* area with an ocular grid (0.0625 mm^2^) from 20 adjacent fields in the cortical area in each specimen. The patients’ information was anonymized and deidentified prior to analysis.

### Colocalization of C3d/CD34 and C3d/α-SMA Detected by Immunofluorescence

To investigate the localization of C3d deposition in the vessels involved, double immunofluorescence staining of C3d/CD34 and C3d/α-SMA was performed. In brief, paraffin sections were deparaffinized and rehydrated as described above. After antigen retrieval was performed as described above, sections were incubated with primary antibodies (rabbit anti-human C3d, 1:1,000 diluted in PBS; mouse anti-human CD34, 1:100 diluted; mouse anti-human α-SMA, 1:100 diluted in PBS) overnight at 4°C. FITC/TRITC-conjugated corresponding secondary antibodies (donkey anti-rabbit IgG, Jackson, United States; donkey anti-mouse IgG, Jackson, United States) were incubated for 30 min at 37°C. The slides were examined by a confocal laser microscope (Olympus viewer 1,000, Japan).

### Statistical Analysis

Continuous variables are expressed as the mean ± standard deviation (*SD*) or median [interquartile range (IQR)]. For the comparisons between different groups, independent samples *t*-test or Mann–Whitney test was used as the data indicated. Non-continuous variables were expressed as proportions and compared using the chi-square test or Fisher’s exact test. Pearson correlation coefficients were used to measure the associations between normally distributed variables. Spearman’s rank-order correlation tests were performed to analyze the correlations among non-normally distributed variables or rank variables. All analyses were conducted using the SPSS statistical software package (version 27.0; Chicago, IL). A two-sided *p*-value < 0.05 was considered statistically significant.

## Results

### Clinical Data of the Cohort

A total of 8,980 patients underwent renal biopsy in our renal division during the period of Jan 2010 to Dec 2018. Among them, 2,121 patients were found to have pathological changes of arterionephrosclerosis, and 2,052 of them were excluded for the concurrent presence of other kidney diseases. The remaining 69 patients with arterionephrosclerosis as their initial and isolated pathological diagnosis were further re-evaluated by renal pathologists and nephrologists for both pathological and clinical features. Finally, 52 patients were enrolled in this study (Figure 1). The mean age of the cohort was 45.0 ± 12.7 years, with 39 (75%) males. The median duration of hypertension was 66 months (IQR: 24–138 months). Forty-one patients (78.9%) were overweight or obese (body mass index ≥ 24 kg/m^2^), and 14 patients (26.9%) had diabetes. The serum creatinine at presentation was 126.1 ± 42.4 μmol/L, with urinary protein excretion 0.75 g/d (IQR: 0.27–1.32 g/d). Serum levels of C3 and C4 were all in the normal range of the 52 patients. Of note, serum C3 levels were positively correlated with hypertension duration (*r* = 0.405, *p* = 0.008) and BMI (*r* = 0.449, *p* = 0.003). Only one patient (1.9%) experienced severe bleeding complication after renal biopsy who received blood transfusion without an invasive procedure. The relevant demographic and clinical characteristics of the patients with arterionephrosclerosis and malignant nephrosclerosis are shown in Table 1.

**TABLE 1:** Clinical features of subjects with arterionephrosclerosis and malignant nephrosclerosis.

Characteristics	Arterionephrosclerosis (*n* = 52)	Malignant nephrosclerosis (*n* = 5)
Age (years)	45.0 ± 12.7	32.6 ± 4.3*
Male, *n* (%)	39 (75.0%)	3 (60.0%)
Hypertension duration (months)	66 (24, 138)	20 (8, 36)
Diabetes mellitus, *n* (%)	14 (26.9%)	0 (0%)
Hyperlipidemia, *n* (%)	35 (67.3%)	0 (0%)
Hyperuricemia/gout, *n* (%)	8 (15.4%)	0 (0%)
Body max index (kg/m^2^)	26.8 ± 3.7	25.2 ± 5.3
SBP (mmHg)	134.5 ± 16.7	195.4 ± 22.5
DBP (mmHg)	84.7 ± 13.9	135.4 ± 23.3
Serum creatinine (μmol/L)	126.1 ± 42.4	294.4 ± 144.1*
eGFR (ml/min/1.73 m^2^)	62.5 ± 24.2	26.40 ± 8.64*
Proteinuria (g/d)	0.75 (0.27, 1.32)	1.14 (0.67, 3.84)
hematuria, *n* (%)	15 (28.8%)	2 (40.0%)
Hemoglobulin (g/L)	139.5 (123.0, 152.0)	118 (106, 131.5)
Platelets (x10^9^/L)	197.5 (172.0, 266.5)	239 (162.5, 294.5)
LDH (U/L)	144.0 (129.0, 170.0)	202 (148.5, 236.0)
Serum C3 (g/L)	1.02 ± 0.19	0.92 ± 0.27
Serum C4 (g/L)	0.27 ± 0.08	0.31 ± 0.07
Calcium channel blockers, *n* (%)	22 (42.3%)	3 (60.0%)
β blockers, *n* (%)	14 (26.9%)	4 (80.0%)
ACEI/ARB, *n* (%)	33 (63.5%)	5 (100.0%)

*p < 0.05 vs. arterionephrosclerosis.

### Histological Studies of the Cohort

**FIGURE 2:**
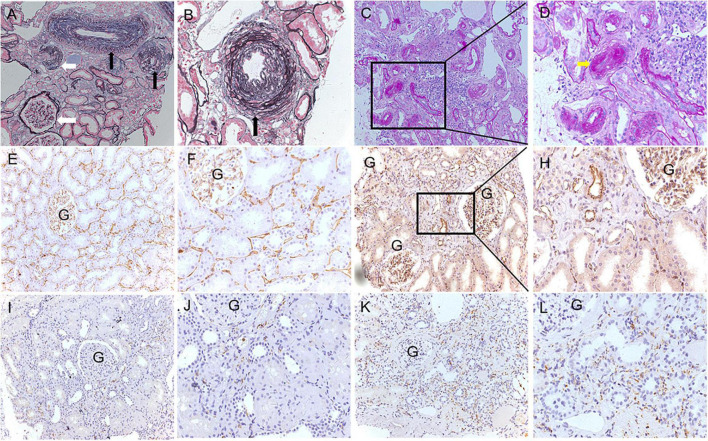
Representative pathological features of arterionephrosclerosis. **(A,B)** Show arteriosclerosis with narrowing of the lumen (black arrows) and ischemic glomerular changes (white arrows) by PASM staining (**A**, 200×; **B**, 400×). **(C,D)** Showed arteriolar hyalinosis with narrowing of the lumen (yellow arrow) by PAS staining (**C**, 200×; **D**, 400×). **(E–H)** Show peritubular capillaries by immunohistochemical staining of CD34 in normal controls (**E**, 200×; **F**, 400×) and arterionephrosclerosis (**G**, 200×; **H**, 400×). **(I–L)** Show infiltration of macrophages by immunohistochemical staining of CD68 in the normal control (**I**, 200×; **J**, 400×) and arterionephrosclerosis (**K**, 200×; **L**, 400×).

The typical histological changes of arterionephrosclerosis are shown in Figure 2. Arteriolar hyalinosis was observed in 21 (40.4%) patients with arterionephrosclerosis. A total of 950 arterioles were evaluated for arteriosclerosis, with the number varying from 4 to 56 arterioles in each specimen. The mean ratio of the inner/outer luminal diameter of the cohort was 0.43 ± 0.05. The proportion of arterioles without arteriosclerosis in each specimen varied from 0 to 86%, with an average of 30 ± 17%. The number of glomeruli in each specimen varied from 7 to 34. Ischemia wrinkling of the GBM and glomerular sclerosis were universal in 76% (IQR: 50.7–94.0%) of the total glomeruli, among which 25% (IQR: 14–45%) with ischemia wrinkling, 31% (IQR: 11.5–43.5%) with segmental glomerulosclerosis, and 9.0% (IQR: 3.0–15.1%) with global glomerulosclerosis. Interstitial fibrosis and tubular atrophy accounts for 10% (IQR: 8–12%) of the interstitial area. As shown in Figure 2, the amount of PTC decreased compared to normal controls, with a mean density of 77.7 ± 31.7 per total area. Additionally, increased infiltration of macrophages in the interstitial was observed in arterionephrosclerosis specimens, with a mean of 37.8 ± 14.4 per area with an ocular grid (0.0625 mm^2^).

### Correlation Between Clinical and Pathological Parameters

The correlations between pathological changes and clinical parameters are summarized in Supplementary Table 2. The ratio of inner-/outer-luminal diameter was negatively correlated with glomerular lesions (*p* = 0.008) and positively correlated with PTC density (*p* = 0.002) and eGFR (*p* = 0.013). PTC density was positively correlated with eGFR (*p* = 0.019) and negatively correlated with glomerular lesions (*p* = 0.005) and interstitial fibrosis (*p* = 0.032). Macrophage infiltration was correlated with interstitial fibrosis (*r* = 0.288, *p* = 0.038).

### Deposition of Complement Components in Renal Specimens

**FIGURE 3:**
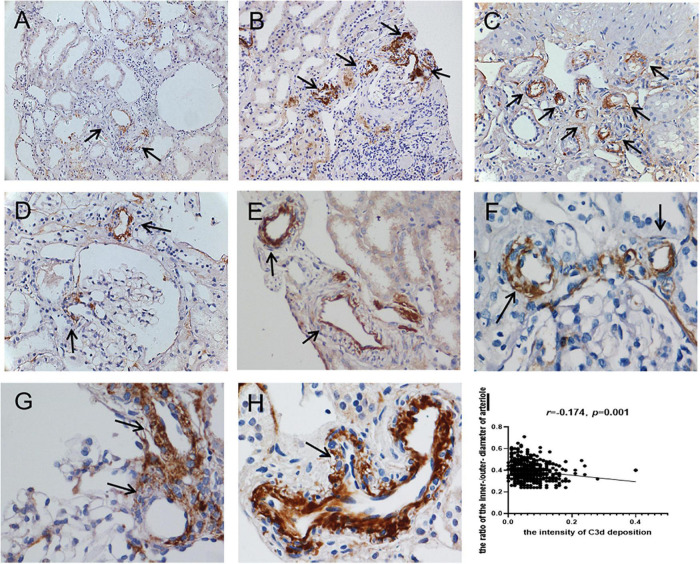
Immunohistochemical staining for C3d deposition in arterionephrosclerosis. C3d was deposited on arterioles with different intensities and patterns (black arrows) (**A–C**, 200×; **D–H**, 400×). **(I)** Showed the correlation between the intensity of C3d deposition and the ratio of the inner/outer diameter of arterioles.

**FIGURE 4:**
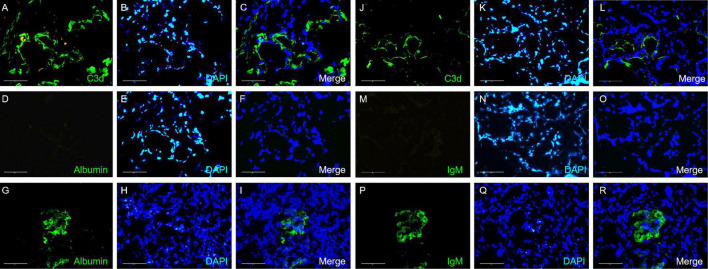
Images of C3d **(A–C,J–L)**, albumin **(D–F)** and IgM **(M–O)** immunofluorescence staining in arterionephrosclerosis. **(A,J)** Showed C3d staining (green) on arterioles. **(D,M)** Showed no staining for albumin and IgM of the same arteriole in **(A,J)**, respectively. **(G–I,P–R)** Showed albumin and IgM staining (green) in positive controls, respectively (Magnification, 400×).

**FIGURE 5:**
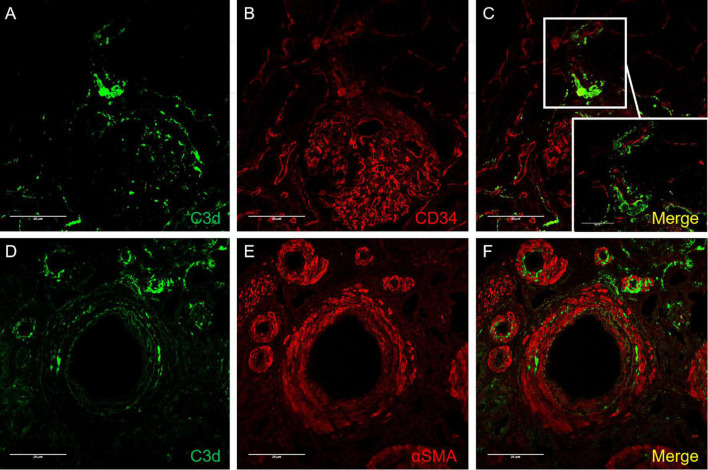
Confocal images of C3d/CD34 **(A–C)** and C3d/α-SMA **(D–F)** immunofluorescence staining in arterionephrosclerosis. **(A,D)** Showed C3d staining (green) on arterioles. **(B)** CD34 staining (red), which was deposited along the endothelial layer of arterioles and glomerular basement membrane. **(E)** For staining α-SMA (red) in arterioles. **(C,F)** Are merged images for C3d/CD34 and C3d/α-SMA, respectively, showing C3d deposition mostly along the endothelial layer with some scattered in the interstitium (Magnification, 400×).

**FIGURE 6:**
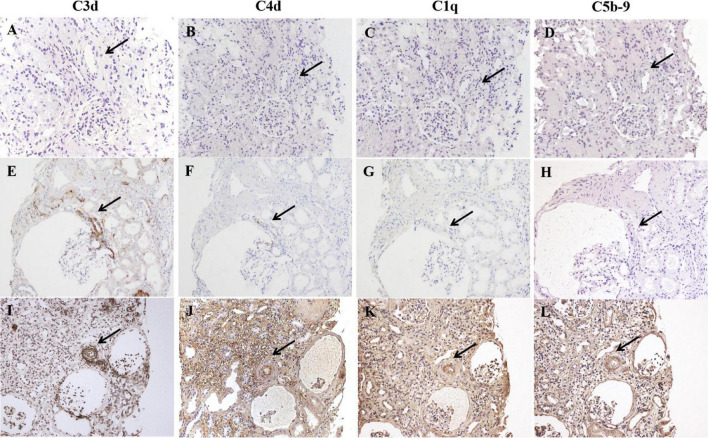
Immunohistochemical staining for C3d, C4d, C1q, and C5b-9 in normal controls **(A–D)**, arterionephrosclerosis **(E–H)** and malignant nephrosclerosis **(I–L)**. The black arrow indicates the same arteriole by different staining. No complement component deposition was found in the normal control **(A–D)**. C3d was deposited with different intensities on arterioles in arterionephrosclerosis, with weak C4d staining observed in C3d-positive afferent arterioles. No C1q or C5b-9 deposition on arterioles was found **(E–H)**. All C3d, C4d, C1q, and C5b-9 were found to be positively deposited along the walls of arterioles in malignant nephrosclerosis (Magnification, 200×).

Seventy-four percent (703/950) of the total evaluated arterioles had C3d staining with various intensities, with the percentage of C3d-positive arterioles ranging from 63.6 to 100.0% in each specimen. C3d was found to be uniformly deposited in hyalinized arterioles and to have an irregular deposition pattern in other involved arterioles (Figure 3). Of note, the ratio of inner-/outer-luminal diameter of arterioles was negatively correlated with the intensity of C3d deposition (*r* = –0.174, *p* = 0.001). To exclude the non-specific deposition of C3d in arterioles, albumin and IgM were detected simultaneously on consecutive sections by immunofluorescence staining. No overlapping staining of albumin or IgM with C3d was observed on arterioles (Figure 4). Using confocal microscopy, C3d deposition was identified to localize in the intima of the arterioles, either along the endothelial layer or scattered in the interstitium (Figure 5). Almost no C4d deposition could be found in arterioles, with occasional positive staining observed in glomerular afferent arterioles and some segments of glomerular capillaries. No C1q or C5b-9 deposition was found on the arterioles in renal arterionephrosclerosis specimens (Figure 6). In contrast, C3d, C4d, C5b-9, and C1q were deposited along the walls of arterioles and/or glomerular capillaries with various intensities in malignant nephrosclerosis (Figure 6). There was no staining of complement components found in normal control specimens (Figure 6).

**FIGURE 7:**
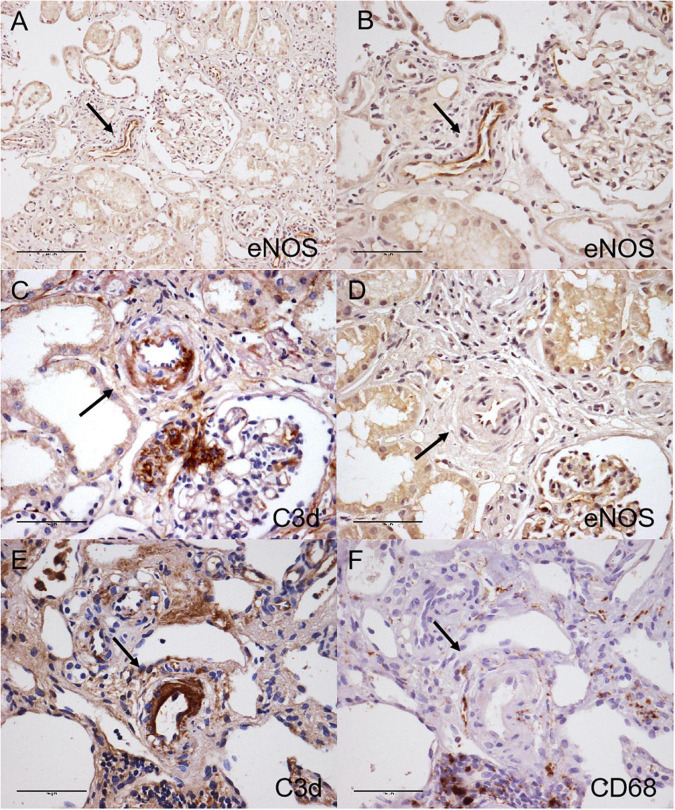
Immunohistochemical staining for C3d **(C,E)**, eNOS **(A,B,D)** and CD68 **(F)** in arterionephrosclerosis. Uninvolved arterioles showed positive eNOS staining along the endothelial layers (**A**, 200×; **B**, 400×). The C3d-positive arteriole with arteriosclerosis showed decreased eNOS staining (**C,D**, black arrow, 400×). **(E,F)** Shows macrophages surrounding C3d-positive staining arterioles (black arrow, 400×).

The uninvolved arterioles showed positive staining of eNOS along the endothelial layers, while C3d staining-positive arterioles showed decreased eNOS staining in arterionephrosclerosis (Figure 7). There was macrophage infiltration in the interstitial in arterionephrosclerosis, with some macrophages surrounding C3d-positive arterioles (Figure 7). The percentage of C3d-positive arterioles was correlated with macrophage infiltration in each specimen (*r* = 0.330, *p* = 0.018).

## Discussion

In this retrospective cohort study, we found a relatively widespread presence of C3d deposition on arterioles in renal biopsy specimens of arterionephrosclerosis. Moreover, the arteriolar deposition of C3d correlated with the degree of arteriosclerosis and interstitial macrophage infiltration, accompanied by decreased endothelial expression of eNOS. These results suggest that complement is activated and may be involved in the development of vascular lesions in arterionephrosclerosis.

Arterionephrosclerosis may progress gradually and develop into end-stage kidney diseases (ESKD). For instance, a study from Japan showed that 117 among 401 patients with biopsy-proven nephrosclerosis showed progression of the disease, which was defined as new-onset ESRD, decreased eGFR by ≥ 50% or doubling of serum creatinine during a median follow-up period of 5.3 years (16). Arteriolosclerosis (media thickening and/or intimal fibrosis) and/or hyalinosis of arterioles are characteristic features of arterionephrosclerosis. These changes are considered initiators of kidney injury, inducing subsequent global/segmental glomerulosclerosis and proportional tubulointerstitial inflammation and fibrosis in arterionephrosclerosis. Ischemia is indicated to play important roles in these processes (17, 18). In the present study, we found that the degree of arteriolosclerosis was positively correlated with glomerular lesions and negatively correlated with peritubular capillary density and eGFR level, supporting the key role of vascular lesions in pathological changes in arterionephrosclerosis.

The pathogenesis of vascular lesions in arterionephrosclerosis has been regarded as the result of increased hydrostatic pressure on the vessel walls in the setting of mild and moderate hypertension for a long time. However, studies of both human and animal models have demonstrated that renal arteriole changes may precede the onset of hypertension, indicating other pathophysiologic mechanisms in the development of vascular lesions in arterionephrosclerosis (19). Recently, accumulating evidence has suggested that aberrant complement activation might accelerate the progression of vascular injury and trigger/aggravate hypertension and hypertension-induced organ damage (14, 15, 20). Clinically, higher plasma C3 levels have been found in hypertensive patients than in normotensive patients. A significant positive correlation between SBP and serum C3 levels was observed in patients with refractory hypertension (21). In a population-based longitudinal study, plasma C3 levels were found to be associated with future blood pressure increases and the development of hypertension. In the present study, we found a positive association between hypertension duration and serum C3 levels. Genetic variants in CFH were found to be associated with blood pressure in a population-based cohort study of Chinese Hans (22). However, pathological evidence of complement activation in arterionephrosclerosis is scarce. C3d is an end-product of C3 cleavage, which binds covalently to cell surfaces as a stable marker of complement activation. In the present study, we observed C3d deposition in arterioles with different staining patterns and intensities in renal biopsy specimens by using immunohistochemical staining. In a previous study, arteriolar C3d staining was described as unspecific entrapment of plasma proteins, as was observed in arterioles with hyalinosis (23). However, we observed C3d deposition not only on arterioles with hyalinosis but also on arterioles without hyalinosis in the present study. No overlap staining of IgM or albumin with C3d in arterioles could be observed. Further analysis showed a positive correlation between the deposition intensity of C3d and the degree of arteriolosclerosis of those arterioles. More importantly, arterioles with C3d deposition had decreased expression of eNOS, a marker of diastolic function of arterioles. These findings suggested the potential participation of complement activation in the vascular lesions of arterionephrosclerosis.

There are three major pathways of complement activation: the classical, lectin, and alternative cascades. All three pathways lead to activation of C3 and its deposition on the target as C3b, which is known as opsonization. We further stained samples for C1q and C4d to reveal the pathway(s) of C3 activation. Neither C1q nor C4d staining was found on the arterioles, suggesting an alternative pathway of C3 activation in arterionephrosclerosis. We found no C5b-9 deposition in the arterionephrosclerosis samples. In contrast, C5b-9, in addition to C3d, were deposited along the renal vasculature and/or glomerular capillary wall in patients with severe hypertension-associated TMA. These findings were in accordance with what Timmermans et al. observed in their study (24, 25). The alternative pathway is a continuously active immune surveillance and effector system operating in circulation and on the cell surface, which is tightly regulated by complement regulatory proteins to prevent self-damage. Based on these findings, we hypothesized that vascular endothelial cells in arterionephrosclerosis might be injured by continuous exposure to hydrostatic pressure and/or pro-hypertensive factors and then detected by AP as a cryptic or neoantigen with subsequent C3b deposition. Given the clinical feature of slow progression of the disease, the production and deposition of C3b on endothelial cells of arterionephrosclerosis may also develop slowly and gradually. Then, the deposited C3b is further cleaved into iC3b and C3d under the regulation of cofactors at the endothelial cell surface, representing a balance between complement activation and regulation without inducing C5b-9 formation. In contrast, C5b-9 deposition reflects overactivation of the complement system. C4d deposition was observed in both Timmermans’ study and our cases with malignant nephrosclerosis. We also found prominent C1q deposition in arterioles and glomerular capillary walls in malignant nephrosclerosis, indicating classical pathway activation. It was reported that severe hypertension-induced shear stress could activate the classical pathway of complement activation (26). We suppose that the classical pathway may act synergically with AP to amplify C3b formation in malignant nephrosclerosis that exceeds the complement regulatory capacity, leading to unrestrained complement activation with C5b-9 formation.

C3a is known to be liberated into circulation as an anaphylatoxin to promote chemotaxis during the activation of C3. We found some macrophages surrounding C3d-positive arterioles in the present study. There was a positive correlation between C3d-positive arterioles and macrophage infiltration. Since inflammation plays an important role in the development of interstitial fibrosis, these results suggested that local C3 activation in the vasculature might contribute to the progression of the disease by recruiting inflammatory cells. In addition, vascular smooth muscle cells are an important constituent cell of the arteriole wall. Bockmeyer et al. reported that arteriolar vascular smooth muscle cells transdifferentiated from a contractile phenotype to a secretory phenotype in benign nephrosclerosis (27). *An in vitro* study showed that C3a could induce exaggerated growth, a synthetic phenotype and angiotensin-II production in vascular smooth muscle cells derived from spontaneously hypertensive rats (28). Whether and how local activation of C3 contributes to vasculature lesions *per ce* in arterionephrosclerosis needs to be studied further.

Our study has the inherent limitations of a retrospective observational investigation. Given the retrospective and descriptive nature of the study, the relationship between C3d staining and vascular injuries must be interpreted cautiously in terms of association rather than causality. This is also applied to the interpretation of other correlation analysis since they are univariate assessment in nature. Additionally, this is a small, single-center retrospective cohort study with a limited number of patients who underwent renal biopsy. Generally, the diagnosis of arterionephrosclerosis is made on the basis of the characteristic clinical features without confirmation by renal biopsy. Therefore, the indications for renal biopsy were not fully standardized, which might bring potential selection bias in our study. Also, sampling error during the biopsy procedure is inevitable, which may also bring bias to the histopathological analysis. Future validation in larger cohorts is warranted.

## Conclusion

In conclusion, our findings provide evidence for complement activation in arterionephrosclerosis. Given the attractive possibility of the complement system as a therapeutic target for inhibiting hypertensive vascular injury, further investigations are needed to verify our findings and clarify the exact mechanism(s) between complement activation and pathogenesis of vascular lesions in arterionephrosclerosis.

## Data Availability Statement

The original contributions presented in the study are included in the article/Supplementary Material, further inquiries can be directed to the corresponding author/s.

## Ethics Statement

The studies involving human participants were reviewed and approved by the Ethics Committee of Peking University First Hospital. The patients/participants provided their written informed consent to participate in this study.

## Author Contributions

YW conceived and designed the research. XC performed the experiments, analyzed the data, prepared the figures, and drafted the manuscript. XY and SW reviewed the renal biopsy. MZ edited and revised the manuscript. All authors approved the final version.

## Conflict of Interest

The authors declare that the research was conducted in the absence of any commercial or financial relationships that could be construed as a potential conflict of interest.

## Publisher’s Note

All claims expressed in this article are solely those of the authors and do not necessarily represent those of their affiliated organizations, or those of the publisher, the editors and the reviewers. Any product that may be evaluated in this article, or claim that may be made by its manufacturer, is not guaranteed or endorsed by the publisher.
