# Supplementary material for: Potential Involvement of Complement Activation in Kidney Vascular Lesions of Arterionephrosclerosis
Source: Front Med (Lausanne). 2022 Mar 31;9:836155. doi: 10.3389/fmed.2022.836155 (PMC9008485; doi:10.3389/fmed.2022.836155)
Supplement: Supplementary file 1 [file Data_Sheet_1.pdf]

Supplementary Table 1 Information of primary antibodies

Antigen	Vendor	Antigen retrieval
IgM	Dako, Denmark	/
albumin	Dako, Denmark	/
C3d	Dako, Denmark	0.4% pepsin 40min, 37℃
C1q	Dako, Denmark	0.4% pepsin 40min, 37℃
C4d	Biomedica, Austria	0.4% pepsin 40min, 37℃
MAC	Abcam, Cambridge, UK	Proteinase K, 10min, 37℃
eNOS	CST, USA	EDTA buffer pH 8.0, 5min, pressure cooker
CD68	Zhongshan Golden Bridge Biotechnology, Beijing, China	EDTA buffer pH 8.0, 2.5min, pressure cooker
αSMA	Abcam, Cambridge, UK	EDTA buffer pH 8.0, 2.5min, pressure cooker
CD34	Abcam, Cambridge, UK	EDTA buffer pH 8.0, 2.5min, pressure cooker

Supplementary Table 2 Correlation between clinical and pathological parameters

variable	Inner-/outer-luminal diameter		glomerular lesions		interstitial fibrosis		PTC density	
	r	*p*	*r*	*p*	*r*	*p*	*r*	*p*
eGFR	0.341	0.013	-0.238	0.089	-0.115	0.417	0.324	0.019
glomerular lesions	-0.364	0.008	/	/	0.361	0.008	-0.385	0.005
interstitial fibrosis	-0.167	0.237	0.361	0.008	/	/	-0.298	0.032
PTC density	0.426	0.002	-0.385	0.005	-0.298	0.032	/	/
